# Substance use and overdose risk: documenting the perspectives of formerly incarcerated persons in the Fraser East region of BC

**DOI:** 10.1186/s12954-021-00525-0

**Published:** 2021-07-28

**Authors:** Celine McCaughran-Contreras, Saranee Fernando, Mike Sikora, Jennifer Hawkins, Marinel Kniseley, Daniel Snyder, Connie Long, James Robson, Amanda Slaunwhite, Amy Salmon

**Affiliations:** 1 University of British Columbia, Vancouver, Canada; 2 CHEOS: Centre for Health Evaluation and Outcome Sciences, Vancouver, Canada; 3 City of Chilliwack, Chilliwack, Canada; 4 Fraser Health, Surrey, Canada; 5 Langley Community Overdose Response, Langley, Canada; 6 Sto:Lo Health Services, Chilliwack, Canada; 7 Resident of Fraser East, Chilliwack, Canada; 8 BC Centre for Disease Control, Vancouver, Canada; 9 University of British Columbia, Centre for Health Evaluation and Outcome Sciences, Vancouver, Canada

**Keywords:** Risk perception, Substance use, Opioid use, Overdose, Incarceration

## Abstract

**Background:**

The relationship between incarceration and risk of overdose has been well-documented in the literature, but few studies document the perspectives of persons at risk of overdose who were incarcerated. This sub-inquiry aimed to understand the experiences of persons with a history of substance use and incarceration in the Fraser East region of BC and how involvement with the criminal justice system affected their drug use and perceived risk of overdose.

**Methods:**

The Fraser East Overdose Response project utilized a community-based participatory action approach that included peer researchers with lived experience in all parts of the research process. This qualitative pilot study aimed to better understand individuals at risk of an unwitnessed overdose in order to prevent deaths and identify effective local responses. A snowball sampling technique was used to recruit persons aged 19 and over who used illicit drugs over the past 3 years in the Fraser East since 2016. In total, 22 participants were interviewed. Of these, 13 participants identified a history of incarceration. Interviews were analyzed using a framework analysis approach.

**Results:**

The perspectives that participants shared revealed that impacts from incarceration are influenced by policies but also highly individualized. Our inquiry found three broader themes, within which were situated differing and sometimes conflicting interpretations and experiences of systemic environments: (1) incarceration was associated with harms and was perceived to increase risk of overdose following release, (2) incarceration was perceived to have limited impact on substance use and overdose risk, and (3) incarceration was associated with a perceived reduction of substance use and overdose risk.

**Conclusions:**

Understanding the complexities of the perceptions of those with lived experience of substance use and incarceration is of importance to better inform interventions in this population. The existing knowledge base urgently requires further inquiry into the intersections between qualitative perspectives, environments and policies, and quantitative outcomes of overdose vís-a-vís correctional institution.

## Background

In April 2016, a public health emergency was declared in response to the rising rates of opioid-related overdoses and deaths in British Columbia. This has largely been attributed to increasing fentanyl in the illicit drug supply, with fentanyl-related overdose deaths rising from 67 to 86% from 2016 to 2020 [1]. The Fraser East region, a rural and semi-urban region within the Fraser Health Authority (FHA), has one of the highest rates of illicit drug overdose death [1]. Chilliwack, a municipality within the Fraser East, experienced a 57% increase in overdose deaths in 2018 compared with 1% increase in all of BC [2]. Understanding the risk factors for overdose in this particular region is important to reducing deaths from overdose in Fraser East and other rural and semi-urban regions that have been greatly affected by the overdose crisis.

The relationship between recent incarceration and risk of overdose has been well-documented in the literature. Previous research has shown that a large proportion of incarcerated persons around the world, and in Canada specifically, report a history of substance use [3, 4]. In the USA, it has been estimated that up to half of incarcerated peoples have a substance use disorder (SUD) [5]. Within Canada, both in provincial and federal correctional settings, injection drug use prior to incarceration was commonly reported, with 2/3 of federal inmates reporting a history of substance use problems [4]. Several studies have found that in the first weeks following release from prison, risk of non-fatal overdose and overdose death is markedly increased, and overdose is one of the leading causes of death related to correctional institutions both during and after incarceration [6–8]. In Canada, there have been fewer studies relating overdose death and incarceration history. A recent BC Centre for Disease Control (BCCDC) Knowledge Update indicated that persons with an incarceration history were 4.1 times more likely to die from overdose-related causes, and between 2015 and 2017, 20% of those who suffered a fatal overdose were previously incarcerated [9]. There are several federal correctional facilities and one provincial correctional facility in the Fraser East region [10, 11]. Thus, the relationship between incarceration and substance use is crucial to explore in the context of the overdose crisis within this region and beyond.

The literature identifies several risk factors and protective factors for overdose following release from prison. Several studies determined a previously diagnosed SUD as an independent risk factor for overdose deaths, as well as psychiatric illness and more than one incarceration [12–14]. In BC, risk factors for fatal overdose death included a SUD diagnosis, especially in combination with mental illness, as well as using opioids for pain management and having multiple chronic illnesses [9]. Another critical factor that has amplified the risk of overdose deaths is the contamination of the drug supply with fentanyl and fentanyl analogues [15–17]. In April 2016, a dramatic rise in the rate of opioid-related overdose deaths led to the declaration of a public health emergency in BC [16], where most fatal overdose events were increasingly fentanyl related. As illustrated in 2020, fentanyl was found in approximately 86% of overdose death cases across the province [9].

As far as protective factors, many studies have identified the availability of harm reduction during incarceration as having a protective effect on overdose deaths post-release. A 2019 systematic review found that opioid agonist treatment (OAT) provided to incarcerated people with opioid use disorder (OUD) reduces overdose incidence and mortality and improved retention rates in treatment services post-release [8]. It also recommended that OAT as well as other harm reduction strategies, including naloxone training and provision, be available before, during and after incarceration to reduce risk of overdose [8]. Although these quantitative analyses certainly inform our understanding of opioid overdose risk following incarceration, the existing knowledge base requires qualitative studies to better understand the intersecting individualized and systemic factors affecting substance use and risk of overdose post-release.

Research exploring the perspectives of previously incarcerated persons who use drugs on their perceptions of overdose risk is limited. A 2012 qualitative study from Denver, Colorado [18], found that poor social support, economic scarcity, and housing and social settings where there was prevalent exposure to illicit drugs were associated with substance use and increased risk of overdose death. Many participants identified decreased tolerance after incarceration or increasing potency of substances as the primary reasons why overdose risk increases post-release [18]. While this highlights risk factors, there is an absence of research since the onset of the current crisis that documents the perspectives of persons at risk of overdose who were recently incarcerated. This sub-analysis of the Fraser East Overdose Response Project aimed to understand the experiences of persons with a history of substance use and incarceration in the Fraser East region of BC and how involvement with the criminal justice system affected their drug use and perceived risk of overdose.

## Methods

### Study Design and Setting

The Fraser East Overdose Response project employed a community-based participatory action approach to develop a research agenda through engagement with various stakeholders in the Fraser East, with the overarching goal of identifying effective place-based interventions to reduce overdose deaths in the region. Stakeholders included persons with lived experience of addiction and overdose, service providers, health administrators, municipal staff and other community members affected by the overdose crisis. Collaboration with these groups helped to highlight research priorities and inform the development of a pilot study that aimed to better understand individual and systemic factors driving increased overdose rates in order to identify effective, patient-centred, place-based responses to prevent overdose deaths in the Fraser East region. The participatory action research (PAR) approach involves peer researchers with lived experience in all parts of the research process and ensures that interventions reflect the contexts and needs of persons who use substances and are at-risk of overdose.

### Participants

We aimed to interview participants who had lived experience of illicit substance use who were or had been at risk of an unwitnessed overdose. Recruitment focused on persons aged 19 and over who used illicit drugs over the past 3 years in the Fraser East. A snowball sampling technique was used in which participants were recruited through activation of the immediate networks of the peer researchers as well as targeted professional and community networks and recruitment posters placed in strategic community locations. In total, 22 participants were interviewed. Of these, 13 participants identified a history of incarceration. Most of the participants interviewed for this pilot study were persons with a history of illicit substance use who had entered a substance use treatment program.

### Interviews

Interviews were conducted in person in public areas or private residences by peer researchers. Peer researchers were trained in research methods and data collection. The interview guide was developed in collaboration with the research team and health administrators. Interviews were audio-recorded and transcribed.

As this was an exploratory pilot study, questions were intentionally kept broad. If the participant’s response indicated incarceration history, follow-up questions were asked. This sub-analysis was informed by all parts of the interviews but specifically focused on questions regarding incarceration and drug use, e.g. “Do you have history of incarceration, being arrested, or any interaction with the criminal justice system?” Follow-up questions included, e.g. “How did being incarcerated affect your drug use?”. Perceived risk of overdose after incarceration was informed by the responses to these questions, as well as questions that more broadly asked about risks related to drug use, e.g. “How would you describe or define risky behaviour within the context of drug use?” and “Do you consider yourself at risk of an overdose?”.

### Analysis

Interviews were analyzed using a framework analysis approach [19]. Two researchers independently reviewed and coded the interviews using N-Vivo Version 12. As themes were identified, they were discussed and summarized in the framework matrix, with rows identifying cases, and columns identifying the major themes that emerged from the coding of the data [16]. Emerging themes and their interpretations were discussed with peer researchers in addition to the other collaborators discussed above.

Demographic information on each participant was informed by a background questionnaire that asked information regarding gender, age, ethnicity, housing and financial situations, interaction with various institutions including correctional facilities, as well as current and past drug use.

## Results

The thirteen participants in this analysis that self-disclosed a history of incarceration included six women and seven men, and the mean age was 35 (range 26–55). Participants described their ethnicity as Indigenous (38%), Caucasian (38%), mixed Indigenous and Caucasian (15%), and other (8%). Participants reported past use of a variety of substances, including opioids (heroin, fentanyl and carfentanyl, and prescription opiates), cocaine, methamphetamines, MDMA, psychedelics, cannabis and alcohol. Five participants reported drug use in the last 3 months, four in the past 6 months and four within the last 3 years.

The perspectives that participants shared regarding the impact of involvement with the criminal justice system on their drug use and perceived risk of overdose revealed three broad thematic relationships: (1) incarceration was associated with harms and was perceived to increase risk of overdose following release (the majority of participants); (2) incarceration was perceived to have limited impact on substance use and overdose risk (some participants); (3) incarceration was associated with a perceived reduction of substance use and overdose risk (a minority of participants). Responses were situated within differing systemic environments and involved individualized and sometimes conflicting interpretations and experiences.

### Theme 1: Incarceration was associated with harms and perceived to increase risk of overdose following release

The majority of participants identified and described several harms associated with incarceration that increased risk of overdose following release. Participants commonly described the harms of forced withdrawal upon entering corrections facilities. For example, one participant (Male, age 38) described the experience:

When I was incarcerated in the last few years and I was addicted to opiates, it was absolute hell going in there, they did not give me any sort of methadone, suboxone. I just sat in there and went through full-blown withdrawals...

Another participant (Male, age 27) also asserted that he felt that withdrawal was the only option they had upon entering prison:

It’s just forcibly stopping you from it. It’s not a decision, it’s more that you have to.

Notably, participants and peer researchers identified differences between federal and provincial correctional institutions, generally describing more robust harm reduction and treatment options available in federal facilities.

Other participants illustrated how incarceration increased their risk of overdose upon release from corrections. Participants explained that incarceration reduced their tolerance and was also related to changes in their drug supply. For instance:

When I haven’t done drugs in so long, I do have a higher risk of overdosing. Doing too much. Or getting something that’s tainted.—(Female, age 26)

One participant described a combination of motive, expectations and a lack of harm reduction information, highlighting that often individuals use similar amounts as they were before they were incarcerated, causing them to overdose:

I think getting clean, and then going out and relapsing and using the same amount you would use before. I think that’s why a lot of people are dying, because their tolerance is way down. Especially guys getting out of jail or getting out of treatment and wanting to use right away. They’re used to a certain amount, and when they go out and relapse that amount, thinking that it’s going to be fine.—(Male, age 31)

The association between decreased tolerance following incarceration and increased risk of overdose was shared by many participants; however, other participants described ongoing access to substances during incarceration and viewed incarceration as having a limited effect on their substance use.

### Theme 2: Incarceration was perceived to have limited impact on substance use and overdose risk

Some participants described a pattern of entering and exiting correctional facilities over time, with these periods of incarcerations’ having little impact on their drug use and behaviours. One participant (Female, age 27) described her criminal justice system experience:

When I was a youth I got into quite a bit of trouble, but I didn’t start going to jail until I was 18. I was constantly in and out. It was because I was wired to heroin and crystal meth, and it led me to do things that normal, sober people don’t do.

Peer analysis emphasized the differences in length of sentencing between provincial and federal institutions, with shorter, cyclical patterns of incarceration appearing more often in provincial institutions.

Another participant (Female, age 32) described how although her drug use ceased during incarceration, it had limited longer-term impact upon release:

Well, it cleaned me up. The second I got out, I remember leaving the Chilliwack courthouse, the first stop I went was to a family friend, an older gentleman, he used to be a neighbour when I lived in Abbotsford. I followed and would always bum money off of him. My first stop was him, and my second stop was the dealer’s.

Additionally, several participants noted that—while access was more difficult—the widespread availability of drugs in federal facilities allowed them to continue their use while incarcerated. For instance, one participant (Female, age 30) explained:

It was consistent. I had drugs all the time…Well it was less, but still there. I’ve done heroin in jail, but only because I brought it with me, and I had a large amount. Me and my roommate were good to go for a couple months…It’s very easy access.

Another participant (Male, age 53) described that although financial barriers exist, drugs were still prevalent in the correctional setting:

It’s there if you can afford it. Probably 5–7 times more expensive than it is on the street. If you can afford it, it’s available, even in prison.

One participant (Male, age 27) described his experience that substances were much less readily available in the provincial institution he was incarcerated in:

You can get weed in jail, you can sometimes get smokes in jail, but anything else you it’s really hard to get in. Especially since I was only in provincial jail. If I was to go to federal, I’d probably find anything at anytime.

This was supported by another participant (Male, age 31) who described his experience in a federal institution:

I went to federal. There’s a lot of drugs in there. When I got that federal sentence, I was at a point where I hit my bottom.

While participants reported varying access to substances according to institutional policies and individual circumstances, these participants did not have to go through withdrawal and could resume similar substance use patterns on release. While the majority of interview participants reported negative or minimal impacts of incarceration, a small minority of participants reported that incarceration reduced their substance use which decreased their risk of future overdose.

### Theme 3: Incarceration was associated with a perceived reduction of substance use and overdose risk

A minority of participants described a perceived reduction of drug use as a result of incarceration. This was mostly related to opportunities for detoxification and/or treatment. For example, one participant (Female, age 32) stated:

I really don’t think I wouldn’t have been able to detox if I could have left. The whole detoxing off of drugs was good because I was literally locked in a box. I had gone to detox a couple times before, when I got to that really rough part, I was gone with all my bags.

While forced withdrawal—particularly in provincial facilities—was experienced detrimentally by some participants, the interviews revealed that it can also be experienced as positive, although possibly by fewer people.

Another few participants described the benefit of harm reduction initiatives such as OAT. A perceived difference in access to OAT and harm reduction was identified in federal versus provincial settings:

I spent my whole time in prison clean. I got onto methadone…When I got in there. They put me on suboxone till they put me on methadone…It did help me. The federal system, they have really good programming. While as the provincial system, they don’t really have programming… I’ve spent lots of time in provincial…It used to be easy to get on suboxone and methadone and all that stuff. I feel now it’s harder.—Male, age 31

Perceptions of how incarceration influences substance use and risk of overdose varied and at times conflicted; uniformly, however, the differences in policies and environments between correctional institutions played a definitive role.

## Discussion

Although the risk of overdose following incarceration has been well documented, our results highlight the importance of including the perspectives of persons with lived experience with substance use and incarceration through qualitative inquiry and the additional inclusion of peer researchers. Our data suggest that among people who use drugs and who have experienced incarceration, there is not a singular perceived relationship between incarceration and overdose risk; instead, several relationships were identified that interfaced with numerous situational, contextual and personal factors influencing these perceptions. In particular, interacting details such as length and patterns of incarceration, motives and expectations relating to drug use and relationships “outside,” access to illicit substances, or differences in harm reduction policies and programming factor prominently in individual experiences.

Several participants described the availability of illicit substances within correctional facilities and that incarceration had little impact on their drug use or behaviours. There were identified barriers (mostly financial) to obtaining substances in prison, but these were mostly viewed as navigable if resources permitted. Participants commonly reported that there were differences between provincial and federal facilities, and in this respect federal facilities were perceived to allow for easier access to substances than provincial facilities. As well, many participants did not report that incarceration changed or affected their drug use behaviours beyond the period of time they were in prison. These findings illustrate the importance of providing harm reduction services to persons who are incarcerated due to the high likelihood of continued substance use while in custody. Needle exchange programs and supervised injection services within prison settings could decrease harm and overdose in community through training and distribution of take-home naloxone and overdose prevention programming [20–22].

Participants also identified the harms related to incarceration, which included both physical withdrawal during incarceration as well as increased risk of overdose after release. Forced withdrawal was viewed as a common occurrence on entering correctional facilities and was described as causing extreme distress. Further, increased risk of overdose was understood as associated with decreased tolerance as a result of incarceration and was perceived as a significant risk following release from prison. Interventions should prioritize harm reduction in the form of OAT during imprisonment to reduce the risk of fatal overdoses due to decreased tolerance [3, 8]. Post-incarceration release is a time when relapse can occur and that transition back to community should support connection to harm reduction services and treatment services [12, 23].

A minority of participants indicated that imprisonment could be beneficial if it allowed for access to treatment and services that persons may not have access to in the community. Participants highlighted the difference between federal and provincial institutions, and provincial facilities were perceived as having less access to programming than federal prisons. Although there were fewer participants who highlighted that incarceration reduced their drug use and decreased their overdose risk, they did suggest that incarceration could provide an opportunity to support individuals with substance use outside of their usual context of drug use. This supports the possibility that correctional facilities could be a space where evidence-informed strategies could support substance users and prevent overdoses [14].

It was evident throughout our data that the perception of incarceration on substance use and overdose risk was highly dependent on different contextual factors that influenced individual experiences. Some of these contextual factors included different facility types, the availability of programming regarding substance use and/or other individual factors. There were definite contrasting perceptions of federal versus provincial facilities, with provincial facilities perceived as being more difficult to obtain drugs in and as having fewer programs for substance use. Programs that were identified as beneficial included OAT including methadone and/or buprenorphine/naloxone; however, participants reported varying perceptions of availability and access to these programs.

Interestingly, participants often identified multiple and sometimes conflicting perspectives on the relationship between incarceration and overdose risk within a single interview. That is, some participants viewed incarceration as both beneficial and harmful, and this depended on the timing and context of the incarceration, where they were incarcerated, and what services were available to them during and after their incarceration. Some individuals even identified that even one incarceration stay could be both harmful and beneficial. This highlights the complexity of the relationship between substance use and incarceration, and that interventions may need to be individual and context-dependent. In addition to safe supply and harm reduction services, peer mentorship programs may help link those who have exited carceral contexts to necessary support services and harm reduction resources and can understand their complex needs [24]. Overall, our data support that intervention needs to be focused on strategies that prioritize harm reduction, needs to provide opportunities for access to services during and after release from prison and needs to be contextualized and individualized.

This study highlights the importance of reflecting on the ways public health messages are communicated at both an individual and community level. The current understanding in public health is that incarceration is associated with an increased risk of overdose; however, the individuals we interviewed did not always perceive this relationship in the same way. This challenges practitioners and public health to ask if we have a complex enough understanding of risk and risk perception.

Finally, it is important for public health initiatives to respond to the current fentanyl crisis in BC. Examining fatal overdoses, a Rhode Island-based study by Brinkley-Rubinstein and colleagues found no significant difference in groups compared by incarceration history, indicating that the impacts of fentanyl contamination in the regional drug supply are deadly among not only those with a history of incarceration [25, 26]. As this is an emerging area of research, we were unable to find similar studies based in BC. However, due to the ubiquity of fentanyl in BC’s drug supply and increasing overdose deaths, public health initiatives strategies have already moved towards improved access to naloxone, enhanced overdose prevention trainings for health professionals and community members and increased surveillance and utilization of overdose data to further inform public health recommendations [9].

There were several limitations to this current inquiry. As a sub-analysis to a larger pilot project, data came from a relatively smaller group of individuals with varying histories of involvement with corrections and incarceration. Thus the data may not be generalizable, and nuanced thematic relationships were more difficult to determine. Most of the participants were also in recovery, and their perspectives may be different from individuals who are actively using substances. Furthermore, in many cases their incarceration history was some time ago and thus may not reflect current practices or realities of individuals who use substances before, during or after incarceration. Their experience with correctional facilities may also not reflect on current policy at correctional institutions. In addition, the recruitment process for this exploratory study may have not been able to reach hidden populations of those who use substances alone and/or experience stigma related to their use and/or incarceration histories.

Given the significant body of evidence related to gendered [27–29] and racialized [30–32] dimensions of overdose and incarceration for drug-related offenses, as well as the impact of continuing colonization on overdose risk and incarceration for Indigenous Peoples in Canada [33, 34], we were surprised to observe that no participants reflected these experiences in their interviews; we saw no differences in responses or emergent themes among participants by gender, race or ethnicity. Although we join colleagues in the understanding that there is a critical need for overdose response policy and practice that addresses gendered, racialized and neo-colonial dimensions of this public health emergency, and on the disproportionate risk of incarcerations among racialized and Indigenous persons, our data offer limited commentary on this.

Future research could also further explore the in-depth experiences and perceptions of persons with more recent experience of incarceration and substance use. Further, the different experiences of those with incarceration at federal versus provincial correctional facilities could be described in greater detail. A larger sample would also help to offer more nuanced insight into the role of incarceration in substance use and risk of overdose following release.

## Conclusions

This inquiry explores the perceptions of persons with lived experience of substance use and incarceration to understand the role that incarceration plays in perceived overdose risk. Broadly, there were three relational themes identified: incarceration was seen as being harmful and increasing overdose risk, incarceration was beneficial and reduced overdose risk by reducing substance use, and lastly, incarceration had little impact on drug use or overdose risk. The interplay of such factors as length and patterns of incarceration, motives and expectations relating to drug use and relationships “outside”, levels of access to illicit substances while in prison, and harm reduction policies and programming varyingly impact individual outcomes and experiences. While we did not have enough data to conclusively identify definitive relationships between various policies, environments and individual perspectives, experiences and behaviours, our sub-analysis reveals the efficacy of qualitative study with peer researchers as well as the need for further inquiry to better inform interventions and public health messaging.

## Data Availability

The qualitative data used and/or analysed during the current study are available from the corresponding author on reasonable request.

## Abbreviations

BCCDC: British Columbia Centre for disease control
FHA: Fraser Health Authority
OAT: Opioid agonist treatment
OUD: Opioid use disorder
PAR: Participatory action research
SUD: Substance use disorder
